# Essential thrombocythemia vs. pre-fibrotic/early primary myelofibrosis: discrimination by laboratory and clinical data

**DOI:** 10.1038/s41408-017-0006-y

**Published:** 2017-12-13

**Authors:** Martin Schalling, Andreas Gleiss, Bettina Gisslinger, Albert Wölfler, Veronika Buxhofer-Ausch, Georg Jeryczynski, Maria-Theresa Krauth, Ingrid Simonitsch-Klupp, Christine Beham-Schmid, Jürgen Thiele, Heinz Gisslinger

**Affiliations:** 1 Department of Internal Medicine I, Division of Hematology and Hemostaseology, Medical University of Vienna, Vienna, Austria; 2 Center for Medical Statistics, Informatics and Intelligent Systems, Medical University of Vienna, Vienna, Austria; 3 Division of Hematology, Department of Internal Medicine, Medical University of Graz, Graz, Austria; 4 Department of Internal Medicine I, Ordensklinikum Linz-Elisabethinen Hospital, Linz, Austria; 5 Clinical Institute of Pathology, Medical University of Vienna, Vienna, Austria; 6 Institute of Pathology, Medical University of Graz, Graz, Austria; 7 Institute of Pathology, University of Cologne, Cologne, Germany

Among several groups of clinicians and hematopathologists a conflict of opinion has been repeatedly expressed concerning the validity of bone marrow (BM) features characterizing myeloproliferative neoplasms (MPNs)^1, 2^. In this regard, controversy is mainly focused on the distinction between essential thrombocythemia (ET) and pre-fibrotic/early primary myelofibrosis (pre-PMF)^3–5^. Although other groups confirmed the characteristic BM features and emphasized the clinical impact to discriminate both MPN subtypes^3, 6–10^, the existence of pre-PMF has been questioned, including clinical usefulness and particularly reproducibility of the corresponding diagnostic guidelines^11^. In this context, it has been criticized that the MPN classification proposed by the World Health Organization (WHO), updated in 2008^12^ and revised in 2016,^13^ was focused on BM morphology as the gold standard of diagnosis. As has been highlighted until now, none of the various mutations identified so far have proven to be specific and therefore cannot be applied for a molecular classification of MPNs and especially not for the distinction between ET and pre-PMF^14^. Given the very different outcome and treatment options in clinical practice, there is an active interest to evaluate whether laboratory or clinical parameters could help to distinguish WHO-defined ET and pre-PMF in patients presenting with thrombocytosis. A first step to elucidate if blood tests could exert a predictive power in patients presenting clinically with an ET-like phenotype was initiated by Carobbio et al^15^. To identify pre-PMF cases mimicking ET, the laboratory parameters for hemoglobin (Hb), white blood cell (WBC) count, and serum lactate dehydrogenase (LDH) level were used in a dichotomized manner, resulting in a step-by-step algorithm. The cutoff values at each step in this algorithm were optimized to produce the desired specificity and sensitivity. The result was that nearly 50% of all patients mimicking an ET-like phenotype could correctly be attributed to the pre-PMF group^15^.

The aim of the present study was to extend and improve this investigation by expanding the algorithm described by Carobbio et al. (the so-called Bergamo algorithm)^15^, so that the discriminatory ability could be raised. The first step was to include splenomegaly as clinical parameter into this algorithm, since it is an important factor setting pre-PMF apart from ET patients^3, 6, 8, 11^. In this regard, left shift in the peripheral blood (presence of single erythroblasts or myelocytes, metamyelocytes, promyelocytes, or myeloblasts) was also tested as a presumptive parameter.

The second and more important approach to this problem was to develop a model which utilizes these parameters in a continuous manner by applying a logistic regression model rather than looking at each parameter in a stepwise order.

This study is based on an Austrian registry diagnosed for MPN according to the 2008 WHO diagnostic criteria between 1985 and 2015, which was created by clinicians and hematopathologists in the Departments of Hematology and Clinical Pathology at the Medical University of Vienna, Austria, in collaboration with other clinical centers throughout Austria. In close cooperation with the local hematopathologists, BM biopsies were centrally re-evaluated under a multi-headed microscope by three of the authors (J.T., I.S-K., C.B-S.) who were blinded to the clinical data except for gender and age at time of biopsy. Final diagnosis according to the 2008 updated^12^ and 2016 revised^13^ WHO criteria, respectively, was made based on the histopathology review, clinical data and (if available) mutation analysis. Only patients with a complete data set, a consistent diagnosis between BM morphology and clinical findings, a platelet count >450 × 10^9^/L and no evidence for masked/early stages of polycythemia vera, including follow-up examinations, were included in this study. The laboratory and clinical data of the 359 available patients (194 WHO-defined ET and 165 pre-PMF) are summarized in Supplementary Table 1.

**Table 1:** Applying the Bergamo algorithm^15^ and a logistic regression model to the Austrian cohort of 359 patients (percentages refer to total number of WHO-ET cases (*n* = 194) in the upper part of the table and of pre-PMF cases (*n* = 165) in the lower part)

WHO-ET (***n*** = 194)	True WHO-ET, ***n*** ^a^	False pre-PMF, ***n***	Undetermined WHO-ET, ***n***
Bergamo algorithm	**88 (45.4%)**	36 (18.5%)	70 (36.1%)
Expanded Bergamo algorithm	**88 (45.4%)**	53 (27.3%)	53 (27.3%)
Regression Model^b^	**150 (77.1%)**	44 (22.9%)	0 (0%)

Pre-PMF (***n*** = 165)	True pre-PMF, ***n*** ^a^	False WHO-ET, ***n***	Undetermined pre-PMF, ***n***
Bergamo algorithm	**78 (47.3%)**	27 (16.4%)	60 (36.4%)
Expanded Bergamo algorithm	**98 (59.4%)**	27 (16.4%)	40 (24.2%)
Regression Model^b^	**128 (76.7%)**	37 (23.3%)	0 (0%)

The regression model is based on continuous laboratory parameters and splenomegaly. The cutoff in the final model between WHO-ET and pre-PMF is set such that sensitivity and specificity are as close as possible

^a^ True pre-PMF means true positive and gives sensitivity; true WHO-ET means true negative and gives specificity (undetermined cases are included in the denominator for calculating percentages)

^b^ Percentages for regression model are corrected for over-optimism

Applying the Bergamo algorithm described by Carobbio et al^15^ to our cohort resulted in 78 identified pre-PMF and 88 confirmed WHO-ET cases (Table 1). In all, 130 patients could not be classified. Expanding the Bergamo algorithm by the potential predictors splenomegaly and left shift (Supplementary Information) increased sensitivity with regard to pre-PMF from 47 to 59%. The number of undetermined cases could be reduced to 93 (Table 1).

While step-by-step procedures like the Bergamo algorithm deliver a prediction in the form of a patient’s direct classification into either ET or pre-PMF (or undetermined), a logistic regression model transforms each set of a patient’s characteristics (continuous laboratory parameters and an indicator for splenomegaly) into a predicted probability that this patient classifies as pre-PMF (in contrast to ET). The information contained in the parameters is thus exploited in an optimal, data-driven way. There are no undetermined cases in this approach. Left shift in erythropoiesis or in granulopoiesis was not included for sake of a parsimonious prediction model since this variable did not further improve discrimination. Appropriate statistical methods were used to correct for the fact that the data used for estimating the model are the same that were used for model validation. Since the data set was of medium size, internal validation procedures were used to fully exploit the available data instead of splitting the data into training and test data. Performance measures such as the AUC were corrected downwards accordingly. The details are reported in the Supplementary Information.

A direct comparison between the Bergamo algorithm^15^ and predictions based on our regression model shows that of the 130 previously unclassified patients, 46.6% could now be classified as pre-PMF and 53.4% as ET. Additionally, 11.0% of the patients previously classified as ET were reclassified as pre-PMF, whereas 22.0% of the patients previously classified as pre-PMF were reclassified as ET (Supplementary Fig. 4). The corrected area under the ROC curve amounts to 0.85.

The distributions of the probabilities of being pre-PMF predicted by the regression model for our patient cohort are shown in Fig. 1 and indicate a good discrimination between diagnosed ET and pre-PMF cases. A cutoff for the predicted probabilities is proposed at 0.438 such that sensitivity and specificity are approximately equal, since we regard the harm of missing a pre-PMF case as equal to that induced by over-treating an ET patient wrongly diagnosed as pre-PMF. Using this cutoff, we achieve the classifications summarized in Table 1.

**Fig. 1:**
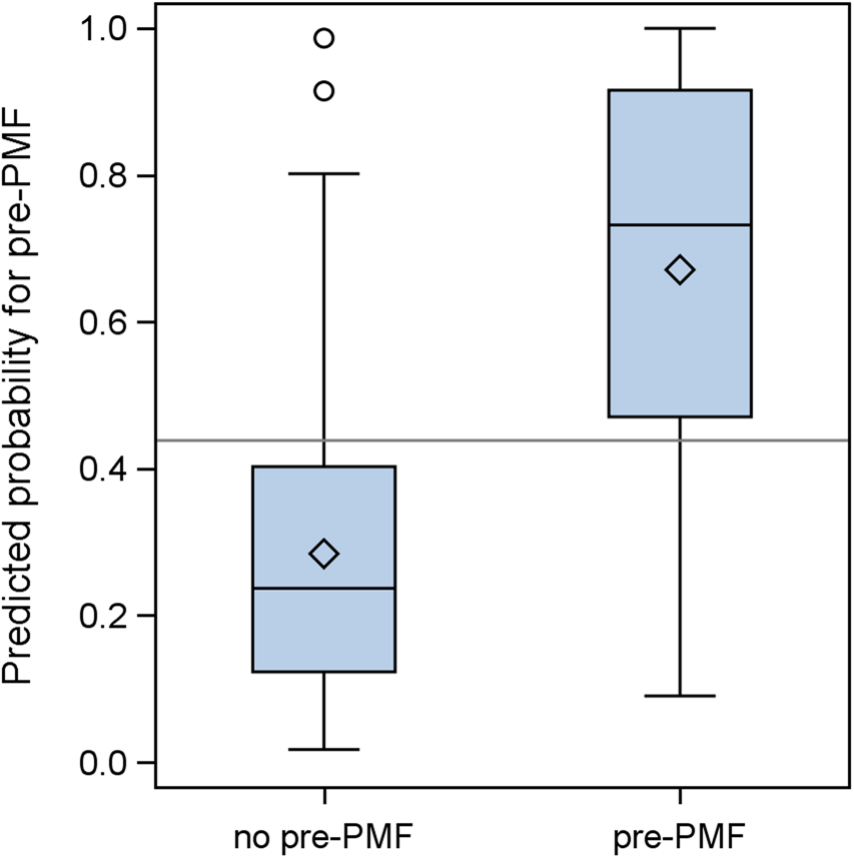
Boxplots of shrunk predicted pre-PMF probabilities Horizontal reference line: cut-off for approximately equal sensitivity and specificity at 0.438

The pre-PMF probability predicted by our regression model can be used as a prediction score ranging from 0 (ET) to 1 (pre-PMF). It is calculated using the following formula:

$${\mathrm{Score = }}\frac{1}{{{\mathrm{1 + exp}}\left(\begin{array}{l}21.01 + 0.249*{\rm Hb}- 0.613*{\rm log}2({\rm WBC})\\ - 2.63*{\rm log}2({\rm LDH}) - 1.04*{\rm splenomegaly}\end{array}\right)}}$$

In this formula, log2 denotes the binary logarithm and 1 is inserted for splenomegaly, if the spleen is palpable or ≥12 cm in length (diameter) in any imaging and zero otherwise. To transform this score into a dichotomous classification rule (ET vs. pre-PMF) a cutoff equal to 0.438 is proposed, which leads to approximately equal sensitivity and specificity. Since this score of differential diagnosis is actually an estimated probability that the patient at hand is pre-PMF (in contrast to ET), a less technical criterion could also be considered, such as managing patients as pre-PMF if the estimated probability of being pre-PMF is at least, e.g., 30%. In such situations, BM biopsy should be requested in order to confirm this diagnosis. Note that the coefficients used in the above formula are shrunk (Supplementary Information) such that its application to cohorts different from ours is made possible.

Admittedly, this formula represents a rather abstract way to look at patient parameters in daily clinical practice. However, using it as a backbone for a web-application, the requested parameters could be entered into a simple interface, which returns a single outcome value (for an example of the application of this formula, see Supplementary Fig. 5).

Using coefficients of discrimination, the relative importance of each ingredient of our formula can be assessed. For all four variables used in the regression model (Hb, log of WBC, log of LDH and splenomegaly), we obtain a corrected coefficient of discrimination equal to 0.36. With a coefficient of discrimination equal to 0.26, LDH is by far the most important single predictor for discriminating pre-PMF from ET, followed by splenomegaly (0.11), Hb (0.07) and WBC (0.06).

These parameters were shown to significantly differ among pre-PMF and ET patients (Hb levels higher in ET than pre-PMF, WBC counts lower in ET than pre-PMF, LDH levels lower in ET than pre-PMF and incidences of palpable splenomegaly lower in ET than pre-PMF)^6, 11^. Our model-based approach, which utilizes exactly those parameters, produces a prediction for all cases of a dataset at hand rather than only sorting them into predetermined categories. In addition, this study demonstrates that a risk score formula based on statistical modeling is able to exploit the information contained in the same predictor variables in a more efficient way.

In conclusion, although, according to the WHO criteria, BM biopsy examination persists to remain an integral part of the final diagnosis, laboratory parameters at presentation may provide clinicians with additional information to suspect pre-PMF in a patient with a presumptive clinical diagnosis of ET. However, in this context it should be underscored that our formula and the proposed cut-off for the resulting score need to be externally validated in other large cohorts of thrombocythemic MPNs patients as well as in prospective clinical trials.

## Electronic supplementary material

Supplemental Information

Supplementary Table 1

Supplementary Figure 1

Supplementary Figure 2

Supplementary Figure 3

Supplementary Figure 4

Supplementary Figure 5
