# Supplementary material for: Essential thrombocythemia vs. pre-fibrotic/early primary myelofibrosis: discrimination by laboratory and clinical data
Source: Blood Cancer J. 2017 Dec 13;7(12):643. doi: 10.1038/s41408-017-0006-y (PMC5802530; doi:10.1038/s41408-017-0006-y)
Supplement: Supplementary file 1 — Supplemental Information [file 41408_2017_6_MOESM1_ESM.docx]

**Statistical analysis**

The distributions of age and laboratory parameters are described by medians and quartiles due to non-normal distributions for some of these variables. Dichotomous variables are described as counts and percentages. Sensitivity and specificity for the Bergamo algorithm and an algorithm expanded by splenomegaly and left shift are calculated by including the undetermined cases into the denominator.

Logistic regression models were used to predict a patient’s verified disease status (pre-PMF vs. ET). In the first model (model 1) indicators for splenomegaly and left shift were used in addition to laboratory parameters dichotomized at the cut-offs employed in the Bergamo algorithm (Hb < 13 for males and Hb < 12 for females, WBC < 7, WBC ≥ 13, LDH < 200). In the second model (final model) laboratory parameters were used as continuous variables in addition to splenomegaly. For WBC and LDH log-transformed values were used as predictors to symmetrize the distribution and stabilize predictions. The predictors used in the model were pre-specified to be splenomegaly and left shift in addition to the laboratory parameters used in the Bergamo algorithm (Supplementary Figure 1). Thus, no variable selection was performed (except that left shift was removed from the final model due to obvious negligibility for the sake of model parsimony). Receiver operating characteristic (ROC) curves were plotted for these models (Supplementary Figure 2) and the area under the ROC curve (AUC) was calculated. Since the models’ discriminatory power was evaluated in the same dataset that was used for model fit, AUC measures were corrected for over-optimism using internal validation based on 1000 regular bootstrap samples.^16^ To assess goodness of fit, a calibration plot contrasting (Supplementary Figure 3) observed versus shrunk predicted pre-PMF probabilities and the Hosmer-Lemeshow test^17^ were used. Predicted probabilities were shrunk using global shrinkage based on leave-one-out cross-validation^18^ and are presented as boxplots for ET and pre-PMF (Figure 1). A formula for calculating a risk score equal to the shrunk predicted pre-PMF probability is proposed. A cut-off for this score is proposed such that sensitivity and specificity are approximately equal. For each of 1000 bootstrap samples a model was fit and the cut-off for shrunk predicted probabilities with equal sensitivity and specificity stored. The proposed cut-off is an average across the 1000 bootstrap cut-off values. Based on this cut-off, percentages of reclassifications between the Bergamo algorithm and the final model were calculated. A correction for over-optimism in reclassification rates was applied in the same way as for the AUC. Finally, coefficients of discrimination^19^ were calculated to quantify, for the set of all predictors and each single predictor, their explanatory power and thus relative importance. It equals the difference of mean predicted probabilities between pre-PMF and ET and can also be interpreted as percentage of variation in the outcome explained by the predictors, similar to the R-square measure in linear regression (though values are known to be typically much lower than 1). The coefficient of discrimination was corrected for over-optimism in the same way as the AUC (see above). All calculations were performed using SAS 9.4 (SAS Inc., 2012; SAS code available upon request to AG). Two-sided p-values ≤ 0.05 were regarded as statistically significant.

**References**

1. Steyerberg, EW, Harrell Jr., FE, Borsboom, GJJM, Eijkemans, MJC, Vergouwe, Y, Habbema JDF. Internal validation of predictive models: Efficiency of some procedures for logistic regression analysis, *J Clin Epidemiol* 2011; **54**: 774-781.
2. Hosmer DW, Lemeshow S, Sturdivant RX. *Applied Logistic Regression,* 3rd edn. Wiley, New York, USA, 2013.
3. Verweij PJM, van Houwelingen HC. Cross-Validation in Survival Analysis. *Stat Med* 1993; **12**: 2305–2314.
4. Tjur T. Coefficients of determination in logistic regression models - A new proposal: The coefficient of discrimination. *Am Stat* 2009; **63**: 366-372.

Figure Legends

Supplementary Figure 1: Depiction of different algorithms utilized to categorize patients based on clinical parameters. (a) Algorithm described by Carobbio et al.^15^ and expanded algorithm. (b) logistic regression model used for final calculations.

Supplementary Figure 2: ROC curves for model 1 and final model. Solid grey reference lines indicate cut-off with approximately equal sensitivity and specificity for the final model.

Supplementary Figure 3: Calibration plot for final logistic regression model comparing observed with shrunk estimated pre-PMF probabilities (linear regression with 95% confidence band).

Supplementary Figure 4: Bar chart showing optimism-corrected reclassification rates between the Bergamo algorithm^15^ (horizontal axis) and our final model (uncl. = unclassified by Bergamo algorithm).

Supplementary Figure 5: Application of the final model formula. Patient 1-048 is confirmed to be WHO-ET, patient 1-007 confirmed to be pre-PMF. The calculations were performed using Excel 14.0 (Microsoft, 2010)
