# Supplementary material for: Essential thrombocythemia vs. pre-fibrotic/early primary myelofibrosis: discrimination by laboratory and clinical data
Source: Blood Cancer J. 2017 Dec 13;7(12):643. doi: 10.1038/s41408-017-0006-y (PMC5802530; doi:10.1038/s41408-017-0006-y)
Supplement: Supplementary file 2 — Supplementary Table 1 [file 41408_2017_6_MOESM2_ESM.docx]

**Supplementary Table 1.** Clinical and laboratory characteristics of the selected patient cohort.

| Parameter | All (n=359) | ET (n=194) | Pre-PMF (n=165) | p-value** |
| --- | --- | --- | --- | --- |
| Age at diagnosis (years)* | 62.5 (48.3-72.3) | 59.5 (45.4-69.3) | 65.2 (51.9-74.8) | 0.002 |
| Hemoglobin (g/dL)* | 14.0 (12.9-14.9) | 14.4 (13.5-15.0) | 13.4 (12.3-14.6) | **<0.001** |
| WBC (10^9^/L)* | 9.4 (7.7-12.3) | 8.7 (7.4-11.0) | 10.3 (8.3-14.5) | **<0.001** |
| LDH (U/L)* | 244 (192.5-313) | 210 (178.3-244.8) | 312 (253-400) | **<0.001** |
| Platelets (10^9^/L)* | 770 (607-962.5) | 727 (621.5-931.5) | 801 (563.5-1000.5) | 0.137 |
| Splenomegaly, n (%) | 100 (27.8%) | 26 (13.4%) | 74 (44.8%) | **<0.001** |
| Left shift, n (%) | 62 (17.3%) | 15 (7.7%) | 47 (28.5%) | **<0.001** |

*median (quartiles); **p-values from univariate logistic regression models (based on log-transformed values in case of WBC, LDH and platelets)

**Abbreviations:** LDH –serum lactate dehydrogenase; Splenomegaly-palpable spleen or ≥ 12 cm in any imaging; Left shift-single peripheral erythro-or myeloblasts
