# Supplementary figures and images for: Essential thrombocythemia vs. pre-fibrotic/early primary myelofibrosis: discrimination by laboratory and clinical data
Source: Blood Cancer J. 2017 Dec 13;7(12):643. doi: 10.1038/s41408-017-0006-y (PMC5802530; doi:10.1038/s41408-017-0006-y)

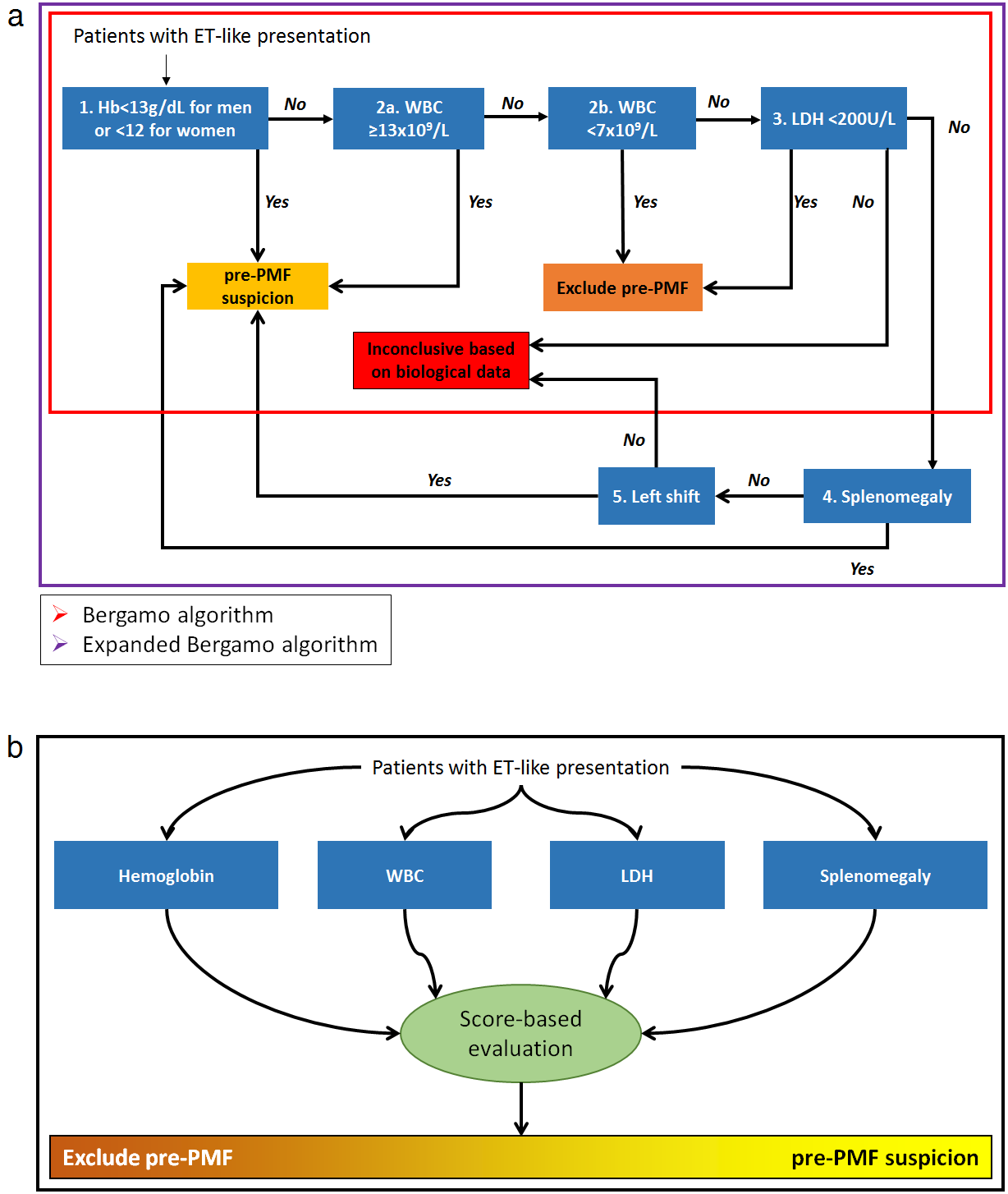

Supplement: Supplementary file 3 — Supplementary Figure 1 [file 41408_2017_6_MOESM3_ESM.png]

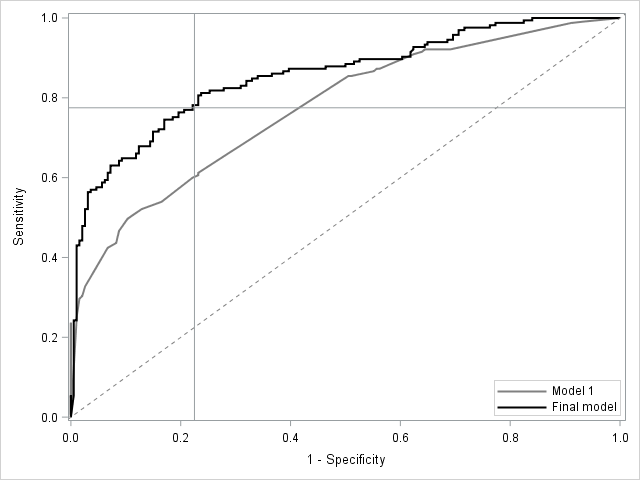

Supplement: Supplementary file 4 — Supplementary Figure 2 [file 41408_2017_6_MOESM4_ESM.png]

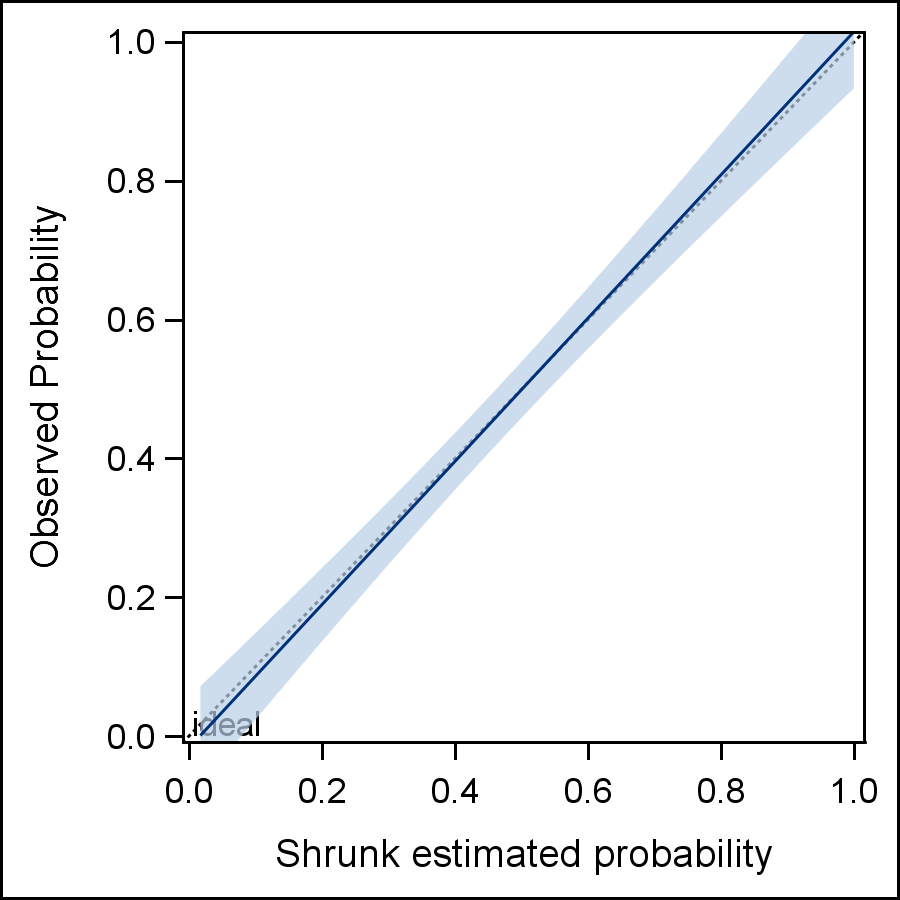

Supplement: Supplementary file 5 — Supplementary Figure 3 [file 41408_2017_6_MOESM5_ESM.tif]

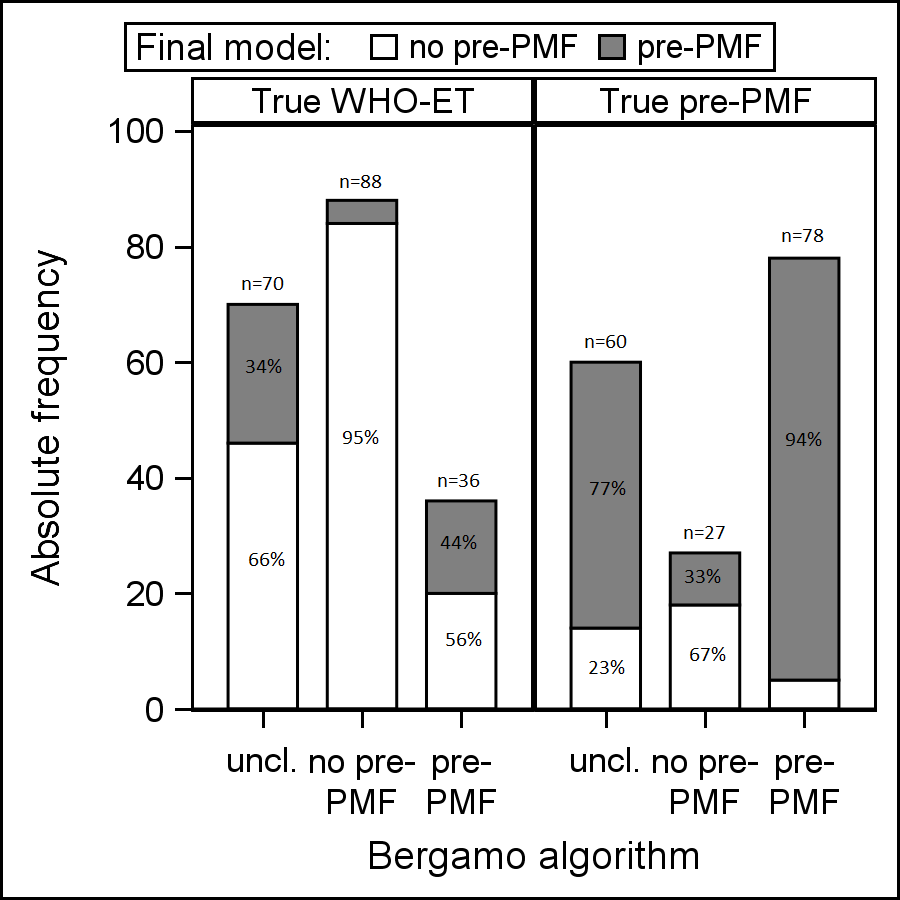

Supplement: Supplementary file 6 — Supplementary Figure 4 [file 41408_2017_6_MOESM6_ESM.tif]

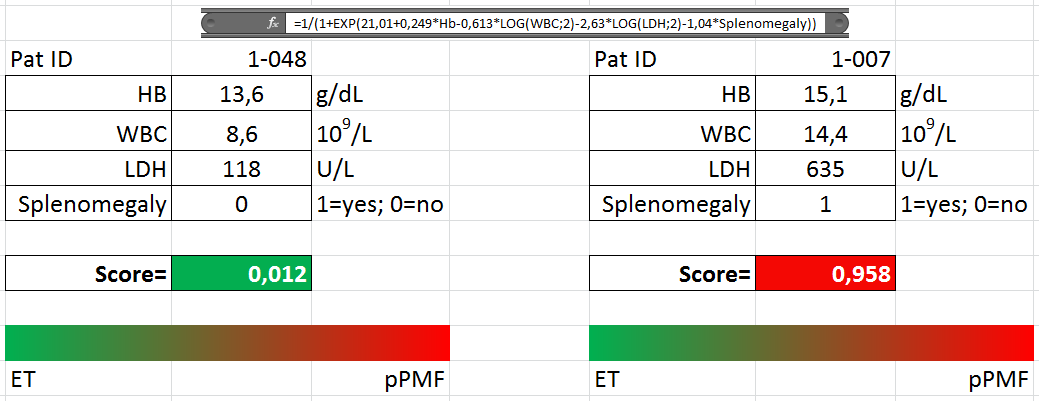

Supplement: Supplementary file 7 — Supplementary Figure 5 [file 41408_2017_6_MOESM7_ESM.png]
